# The Dimension of Time in Host-Microbiome Interactions

**DOI:** 10.1128/mSystems.00216-18

**Published:** 2019-02-19

**Authors:** Giulia T. Uhr, Lenka Dohnalová, Christoph A. Thaiss

**Affiliations:** a Microbiology Department, Perelman School of Medicine, University of Pennsylvania, Philadelphia, Pennsylvania, USA

**Keywords:** microbiome, short-term and long-term interventions, temporal dynamics

## Abstract

The intestinal microbiota contains trillions of commensal microorganisms that shape multiple aspects of host physiology and disease. In contrast to the host’s genome, the microbiome is amenable to change over the course of an organism’s lifetime, providing an opportunity to therapeutically modulate the microbiome’s impact on human pathophysiology. In this Perspective, we highlight environmental factors that regulate the temporal dynamics of the intestinal microbiome, with a particular focus on the different time scales at which they act. We propose that the identification of transient and intermediate states of microbiome responses to perturbations is essential for understanding the rules that govern the behavior of this ecosystem. The delineation of microbiome dynamics is also helpful for distinguishing cause and effect in microbiome responses to environmental stimuli. Understanding the dimension of time in host-microbiome interactions is therefore critical for therapeutic strategies that aim at short-term or long-term engineering of the intestinal microbial community.

## Abstract (precis)

The intestinal microbiota contains trillions of commensal microorganisms that shape multiple aspects of host physiology and disease. In contrast to the host’s genome, the microbiome is amenable to change over the course of an organism’s lifetime, providing an opportunity to therapeutically modulate the microbiome’s impact on human pathophysiology.

## PERSPECTIVE

**FIG 1:**
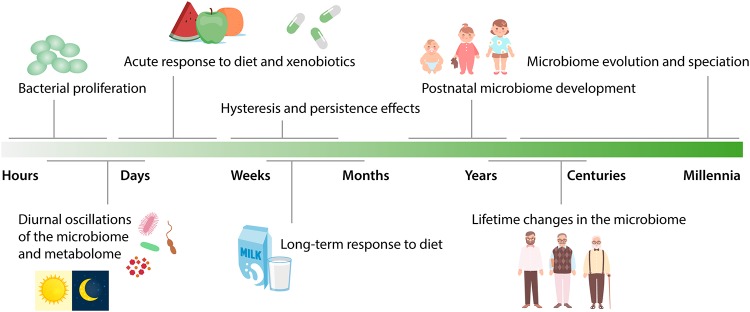
Time scales of microbiome dynamics. Schematic overview of major factors impacting the microbiome and the time scales at which they act.

The intestinal microbiome has been recognized as a major component of the human endocrine system whose digestive and secretory activities profoundly influence most other organs of the human body (1, 2). As such, the microbiome’s activity is critical for human health, and aberrations in the function of the microbiome are involved in the pathophysiology of multiple diseases, ranging from inflammatory and metabolic to neoplastic and neurological diseases (3–7). Consequently, intensive research efforts are currently focused on modulating the intestinal microbiota to promote health and to prevent or counteract disease (8). A property of the intestinal microbial ecosystem that needs to be well understood for therapeutic modulations to be effective is its dynamic behavior over time. In the following sections, we will provide a broad overview of some of the knowns and unknowns regarding the dimension of time in microbiome research. The temporal dynamics of the microbiome important for host physiology range from the scale of hours, reflecting the shortest bacterial generation times, to the scale of centuries and millennia, during which intestinal microorganisms have coevolved with their host (Fig. 1).

## HOURS: BACTERIAL PROLIFERATION AND DIURNAL RHYTHMS

The fastest processes studied in the context of the gut microbial ecosystem have been bacterial proliferation and infection by bacteriophages, some of which occur at the subhour scale (9). Microbiome-wide proliferation rates have been assessed by metagenomic inference, implying widespread hour-scale changes in genomic abundances (10, 11).

A particular organizing principle of such short-term dynamics is diurnal oscillations. The relative and absolute abundances of intestinal bacteria, their biogeography in the gastrointestinal tract, and the intestinal metabolite profile undergo hour-scale oscillations over the course of a day (12–19), indicating that the microbiome is highly dynamic and contains autocorrelative features (self-similarity over a time course) even at short-term intervals. These daily microbial fluctuations are strongly influenced by the time and type of food intake (13, 15, 16, 20). The fact that the intestinal microbiota is fluctuating in composition and function at such short time scales may affect numerous short-term responses of the intestinal community to foreign elements entering the gastrointestinal system, including pathogens, xenobiotics (substances that are foreign to the body, such as drugs and environmental toxins), and dietary nutrients (18, 21).

It should be noted that these conclusions about daily oscillations of the gut microbiome are mostly based on mouse studies, and that the majority of human data is so far only available from a limited number of stool samples and only on the level of relative taxonomical composition. Recent studies have assessed the concept of diurnal microbiome oscillations in human saliva, a tissue more accessible for repeated sampling over the course of a day. These studies have likewise documented oscillatory behavior in taxonomic composition and the dependency of this diurnal property on the timing of food intake (22–24).

The realization that microbiome taxonomy and metabolic function are distinct during day and night at various body sites provides an example for how a better understanding of temporal microbiome behavior can improve the design of analytical studies and therapeutic interventions that harness the optimal time of day for microbiome modulation. For instance, delivery of drugs or probiotics at the ideal time of day in terms of microbiome biotransformation reactions or colonization resistance harbors great optimization potential for already existing therapies.

## DAYS: ACUTE RESPONSES TO INGESTED FOOD AND XENOBIOTICS

Diet and ingested xenobiotics strongly shape the intestinal microbial community and offer opportunities for noninvasive strategies for microbiome modulation. The preponderance of evidence suggests that marked alterations in diet can perturb the taxonomical configuration of the microbiota within a few days.

One of the first studies that longitudinally measured daily responses of the microbiome to environmental perturbations found rapid adaptations of the intestinal microbial community to changes in diet (25). A controlled study of healthy volunteers consuming defined diets showed that microbiota composition shifts caused by dietary intervention were effective already within 24 h (26). Short-term consumption (4 days) of diets based entirely on either animal or plant products was sufficient to introduce distinct community-wide alterations of the intestinal microbiota, coupled to rapid changes in the concentration of intestinal short-chain fatty acids (26).

Microbiome responses to other ingested substances can be similarly rapid. In particular, it has been shown in humans and animal models that antibiotics dramatically alter the gut microbial composition within a few days. Within 4 days of broad-spectrum antibiotic treatment, microbiome diversity plummets, resulting in long-lasting deviations of community structure from the initial composition even several months later (27, 28).

It is important to note that these findings have primarily been based on sampling of the stool microbiome, which is distinct from the microbial communities in upper areas of the small and large intestines (29). The time scales at which different regions of the gastrointestinal tract respond to dietary and xenobiotic perturbations, and the degree of synchronization or codependency between different anatomic regions, remain to be investigated.

## WEEKS AND MONTHS: DIETARY PATTERNS

Diet also influences the composition and function of the microbiota on longer time scales. In particular, long-term dietary patterns kept over several months are a strong factor influencing the overall composition of the microbiota (30). As such, stable exposure to external factors is a critical determinant of long-term microbiota behavior—a concept that is important for using microbiome features as biomarkers for clinical outcome and prognostic or diagnostic disease indicators.

In addition, several recent studies have uncovered additional properties of microbiome temporal dynamics: hysteresis (dependence on past stimuli) and persistence (prolonged effect after disappearance of the initial stimulus). Repeated exposures to alternating diets in mice lead to a hysteresis effect, whereby the microbiota retains characteristics from the previous cycle of exposure in each subsequent cycle (31); hence, the return to the original configuration is impeded with each cycle. Similarly, episodes of low-fiber or high-fat diet lead to long-term persistence of specific microbial features despite the return to normal dietary conditions (32, 33).

Several studies have revealed an annual cyclic reconfiguration of the microbiome that reflects the seasonal availability of different types of food. Evidence for this phenomenon has been found in squirrels (34), wild great apes (35), and human indigenous hunter-gatherer populations (36).

Interestingly, succession of microbial community assembly in natural environments likewise spans time periods of weeks to months, as has been documented after resolution of enteric infection (37) and during mammalian corpse decomposition (38).

## YEARS: LIFETIME MICROBIOME CHANGES

While the microbiome remains relatively stable throughout adult life (39), two periods of life are particularly unstable with regard to the overall taxonomy of the intestinal microbial community: early life and old age. In the early years of life, there are different factors influencing the development of the microbiome, including the delivery mode, breastfeeding, and the introduction of solid food (40, 41). After around 3 years, the relative proportions of microbial taxa remain mostly stable, but the microbiome composition can be altered over time by changes in diet as well as by antibiotics (39, 42), which may even have an intergenerational effect (32, 43). This early susceptibility of the microbiome to community perturbations is physiologically meaningful, since long-lasting detrimental effects on host health have been documented in cases of early-life microbiome disruption by antibiotics or caesarian section (44–46).

In the elderly, the microbiome composition was found to feature a distinct taxonomy compared to the average microbiome of healthy adults (47, 48) This difference has been associated with several age-related processes, such as weakened gut barrier integrity, intestinal pro- versus anti-inflammatory balance, immune and cardiometabolic health, reduced mobility, hospitalizations, and use of medications (47–50). However, the direction of causality is unclear, and dissimilar results have been reported for elderly populations at different geographical locations, highlighting the necessity for strictly controlled studies to discern age-related from confounding environmental factors (51).

## CENTURIES AND BEYOND: MICROBIOME-HOST COEVOLUTION

The essential role of the microbiome for human health makes it particularly interesting to consider long-term microbiome changes throughout human evolution. Given the technical challenges associated with archeological human microbiome samples and their sparsity, studies probing the evolution of the microbiota have so far relied on comparisons of humans with phylogenetically related species. A major question regarding the long-term evolution of the human microbiome is whether its ability to synthesize key components of human metabolism, such as vitamins, and to digest complex carbohydrates have undergone major alterations following the agricultural and later the industrial revolution.

Comparative analysis of microbiomes from African apes demonstrated that host phylogenetic divergence scales with microbiome divergence. Interestingly, compositional changes have accelerated during human evolution and have deviated from the formerly clock-like pace during African ape diversification (52). This analysis further revealed that major taxa of the commensal microbiota have cospeciated with the hominid host, i.e., they have been maintained exclusively within an evolutionary lineage for hundreds of thousands of generations and have diversified alongside host evolution (53).

Experiments in mice have indicated that the microbiota is primarily vertically transmitted, but that there are certain taxa that undergo horizontal transmission, particularly those that potentially cause pathogenicity to the host (54).

Recent studies have shown that within-host evolution of the microbiome can also occur at rapid time scales, even within the life span of a host. For instance, bacterial phenotypes are rapidly varied by invertible promoters upon selective pressure (55). Other facilitators of such rapid within-host evolution of commensal bacteria are homologous recombination and *de novo* mutations (56, 57).

## OUTLOOK

The last decade of microbiome research has brought numerous surprising insights into the diverse impact of the intestinal bacterial community on seemingly unrelated aspects of host physiology, from hepatic metabolism to blood-brain barrier function (58–60). Consequently, microbiome modulatory strategies are of vast academic and medical interest. Surprisingly little is known about the requirements for timing and duration of such interventions, although these factors are probably fundamental for the success of any treatment strategy. While the major principles underlying the temporal dynamics of the intestinal microbiome still need to be unraveled, a number of paradigms have emerged from recent studies.

First, the microbiome is exquisitely susceptible to change early in life. Thus, the ecosystem is particularly vulnerable to perturbation during early childhood, resulting from skin contacts, mode of delivery, and neonatal diet. On the other hand, this period also offers unique opportunities for therapeutic interventions with long-lasting effects. Likewise, the microbiome might be more susceptible to taxonomic alterations in the elderly, although more experimental and empirical data are required to support this notion.

Second, the long-term evolution of the human microbiome seems to be accompanied by loss of species diversity, at least in the last century (61). This loss of species diversity might be disadvantageous for human health, and strategies should be considered of how to rediversify the human microbiome.

Third, the microbiome is amenable to long-term changes. These changes are provoked by persistent lifestyle changes, including long-term dietary patterns and geographical localization. Such long-term approaches might ultimately turn out to be the most optimally suited interventions to engineer the human microbiome for health improvements.

Fourth, on intermediate time scales, the intestinal microbiome features hysteresis and “memory-like” properties. As such, prior exposures to environmental stimuli leave long-lasting signatures in the taxonomic composition and functional properties of the microbiome which influence the response to subsequent stimuli.

An extension of this list of insights into the temporal dynamics of the human microbiome is an essential goal of microbiome research in the years to come. To achieve this, the microbiome research community needs to embrace strategies to incorporate the factor of time in study designs and sampling frequencies. To resolve the spectrum of time scales discussed here, ideal protocols would include sampling frequencies of hours to days (62). The power of such protocols is exemplified by the identification of decaying autocorrelation, i.e., loss of self-similarity over time, in highly time-resolved data sets of the commensal microbiome (28, 63). Such sampling strategies would also enable deeper insights into the phenomenon of rare anaerobe blooms that seem to occur independently of dietary patterns (63).

A higher sampling frequency would facilitate yet another goal of the microbiome research community: the quest to distinguish correlation from causation in human studies. Enhanced temporal resolution in microbiome intervention studies will highlight transient and intermediate stages in microbial community responses to a given perturbation. Identifying and characterizing these intermediate stages will be essential not only to better understand ecological dynamics in the gut but also to distinguish cause and effect in the transition of the microbiome from one state to another. These temporal chains of cause and effect, in turn, are exactly the type of insight that we need in order to design rational therapeutic interventions targeted at the microbiome. As the tools now exist, microbiome research is ready for a venture into the dimension of time.
